# Activation of disease resistance against *Botryosphaeria dothidea* by downregulating the expression of *MdSYP121* in apple

**DOI:** 10.1038/s41438-018-0030-5

**Published:** 2018-05-01

**Authors:** Xiaowen He, Yanhong Huo, Xiuxia Liu, Qianqian Zhou, Shouqian Feng, Xiang Shen, Baohua Li, Shujing Wu, Xuesen Chen

**Affiliations:** 1 State Key Laboratory of Crop Biology, College of Horticulture Sciences and Engineering, Shandong Agricultural University, Daizong Street No. 61, Tai’an, Shandong 271018 China; 2 College of Plant Health and Medicine, Qingdao Agricultural University, Changcheng Road No. 700, Qingdao, Shandong 266109 China

## Abstract

In plants, the vesicle fusion process plays a vital role in pathogen defence. However, the importance of the vesicle fusion process in apple ring rot has not been studied. Here, we isolated and characterised the apple syntaxin gene *MdSYP121*. Silencing the *MdSYP121* gene in transgenic apple calli increased tolerance to *Botryosphaeria dothidea* infection; this increased tolerance was correlated with salicylic acid (SA) synthesis-related and signalling-related gene transcription. In contrast, overexpressing *MdSYP121* in apple calli resulted in the opposite phenotypes. In addition, the results of RNA sequencing (RNA-Seq) and quantitative real-time PCR (qRT-PCR) assays suggested that *MdSYP121* plays an important role in responses to oxidation–reduction reactions. Silencing *MdSYP121* in apple calli enhanced the expression levels of reactive oxygen species (ROS)-related genes and the activity of ROS-related enzymes. The enhanced defence response status in MdSYP121-RNAi lines suggests that syntaxins are involved in the defence response to *B. dothidea*. More importantly, we showed that MdSYP121 forms a soluble N-ethylmaleimide-sensitive factor attachment protein receptor (SNARE) complex with MdSNAP33, and the complex may participate in regulating resistance to *B. dothidea*. In conclusion, by regulating the interaction of SA pathway and oxidation–reduction process, *MdSYP121* can influence the pathogen infection process in apple.

## Plant defense: Regulating resistance to ring rot

A gene with multiple functions in defense against apple ring rot may provide a target for breeding apple varieties resistant to this devastating and enigmatic fungal disease. In China, the world’s greatest producer of apples, apple ring rot poses a severe challenge to growers. A Chinese team led by Shujing Wu and Xuesen Chen, of Shandong Agricultural University, explored the role of a gene, *MdSYP121*, thought to provide resistance to ring rot. A combination of genetic, genomic, and transgenic experiments showed that *MdSYP121* does indeed confer resistance to the disease, and may do this both by regulating hormonal pathways of defense and by stimulating enzymes involved in defensive oxidation/reduction reactions. Overall, their results suggest potential foci for breeding programmes aimed at reducing the susceptibility of apple cultivars to the ring rot fungus.

## Introduction

During the long-term process of evolution, plants have evolved a secretory pathway, which is critical for biosynthetic and endocytic trafficking to the plasma membrane (PM) and vacuole^1^. Secretory membrane trafficking mechanisms have recently been shown to be involved in a variety of plant-specific processes, including plant development, tropic responses and pathogen defence^2,3^. Most endoplasmic reticulum-localised proteins and ER-resident chaperones of the secretory pathway and many antimicrobial compounds are destined for the apoplast or various organelles upon pathogen attack and penetration^4,5^.

In plants, fusion of the endomembrane system is important and rather difficult. During the evolution of eukaryotes, a specialised class of proteins, the soluble N-ethylmaleimide-sensitive factor attachment protein receptors (SNAREs), were formed. These receptors function as mediators of fusion between vesicles and target lipid bilayers^3,6^. Based on the occurrence of either a conserved glutamine or arginine residue in the centre of the SNARE domain, SNAREs can be grouped as target site-localised Q-SNAREs (Qa, Qb, Qc and Q(b+c)) and vesicle-residing R-SNAREs^7,8^. SNAREs drive membrane merging by forming SNARE complexes via intermolecular interactions among vesicles and target subcellular SNAREs. A typical SNARE complex involves distinct types of SNARE proteins (Qa+Qb+Qc or Qa+Q(b+c)) and one R-SNARE polypeptide that together contribute to a four-helix bundle of intertwined SNARE domains^9^.

SNARE-domain proteins represent a fundamental role in biotic defence against fungal pathogens. At least two SNARE protein-mediated exocytosis pathways appear to drive the directional or non-directional secretion of antimicrobial compounds, including defence-related proteins and cell wall building blocks, into the apoplastic space to terminate the infection of fungi or bacteria^10–12^. On one hand, plants exhibit an active resistance mechanism to combat extracellular infection. Silencing a specific PM syntaxin, *NbSYP132*, in *Nicotiana benthamiana* impairs the accumulation of a subset of pathogenesis-related (PR) proteins in the cell wall. *NbSYP132* also contributes to basal and salicylate-associated defence. These results indicate that SYP132-mediated secretion is an important component of resistance against bacterial pathogens in plants^11^. In addition, a SNAP25 homologue is required for pathogen resistance. Mutants of the barley *MLO* gene are fully resistant to powdery mildew; silencing the barley *HvSNAP34* gene, a *SNAP25* homologue, revealed increased fungal entry rates in the *mlo* genotype^10^. On the other hand, cell-autonomous immunity is widespread in plant–fungal interactions and terminates fungal infection after pathogen entry^12^. Plants exhibit resistance by forming SNARE secretory complexes, and pathogen-induced subcellular dynamics enable the execution of immune responses by focal secretion^12^. The AtSYP122 syntaxin functions in exocytosis during defence responses to strengthen the cell wall and prevent penetration by microbes^13,14^. In addition, syntaxins can regulate heavy vesicle traffic towards the site of attempted infection when plants are subjected to fungal attack^14^.

SNARE syntaxin 121 (SYP121) is one of the most important SNARE proteins. SYP121, which is a Qa-SNARE protein, cycles continuously between the PM and endosomes^15^ and has a fundamental role in plant defence^10^. Importantly, SYP121-dependent disease resistance acts in vivo mainly by forming SNARE complexes together with the SNAP33 adaptor and a subset of vesicle-associated membrane protein (VAMP) subfamily members, VAMP721/VAMP722^12^. In Arabidopsis, *SYP121* is an important element in resistance to non-host powdery mildew fungi. In addition, the barley *ROR2* gene, a functional homologue of *AtSYP121*, is required for basal penetration resistance against *Blumeria graminis* f. sp. *hordei* (*Bgh*)^10^. A mechanistic link has been demonstrated between non-host resistance and basal penetration resistance in monocotyledons and dicotyledons^10^. AtSYP121 is encoded by *PEN1*^10^. The Arabidopsis mutant *pen1-1* caused delayed formation of localised cell wall appositions (so-called papillae), resulting in increased *Bgh* infection at attack sites^16^. In addition, using brefeldin A (BFA) showed that interference in endosome recycling to the PM both severely inhibited the SYP121 focal accumulation at fungal entry sites that accompanied delayed callose deposition and compromised defence against powdery mildew fungi^17^. However, Zhang et al. reported that *SYP121* and its homologue *SYP122* are negative regulators of salicylic acid (SA) signalling and programmed cell death (PCD)^18^. The enhanced SA and jasmonic acid (JA) signalling levels and PCD in the syntaxin double mutant *syp121-1 syp122-1* contributed to improved disease resistance against the virulent powdery mildew fungus *Erysiphe cichoracearum* and the bacterial pathogen *Pseudomonas* DC300 in an SA-independent manner^18^. These results suggested that SNAREs employs different mechanisms to regulate both the initial penetration resistance process and the subsequent immune signalling process; these processes differentially contribute to disease resistance. The PM-localised syntaxin-related 1 (*StSYR1*) and *StSNAP33* genes in potato are homologues of Arabidopsis *AtSYP121* and *AtSNAP33*, respectively. The resistance of both StSYR1-RNA interference (RNAi) and StSNAP33-RNAi plants increased and was correlated with the constitutive accumulation of SA and PR1 transcripts, and these plants displayed an early senescence-like phenotype showing chlorosis and necrosis. In addition, downregulation of *StSYR1* led to enhanced resistance against *Phytophthora infestans* and a cell death response at the site of infection^19^.

Apple is one of the most widely cultivated fruits in the world. China has the largest planting area and is the greatest producer of apples worldwide. Apple ring rot is one of the most devastating diseases in China and greatly affects the production of apple;^20^ this disease occurs mostly in the Circum-Bohai-Sea region (Shandong, Hebei and Liaoning provinces)^21^. Since the 1980s, in conjunction with the widespread planting of Fuji cultivars, the area of apple ring rot has increased in eastern China^20^. This disease, which is caused by the fungal pathogen *Botryosphaeria dothidea*, infects both fruits and branches^20^. The resistance mechanism of apple ring rot is very complex, and few studies have examined the molecular mechanism of apple resistance to *B. dothidea* infection. In the present study, based on RNA-Seq results, the *MdSYP121* gene was isolated in apple. The results of a series of genomic, genetic and transgenic experiments suggest that *MdSYP121* plays an important role in *B. dothidea* resistance by affecting the oxidation–reduction process in apple. More importantly, we showed that a SNARE complex composed of MdSYP121 and MdSNAP33 may play an important role in pathogen resistance. Together, these results increase the knowledge regarding the biological roles of SNARE proteins in disease resistance and improve our understanding of the pathogenesis of *B. dothidea*. This study hints at a useful disease management strategy and is helpful for breeding resistance to apple ring rot.

## Materials and methods

### Cultivation and treatment of plants

Tissue-cultured calli of the apple cultivar ‘Orin’ were subcultured under the basic growth conditions of 24 ± 0.5 °C and 24 h of darkness (at a relative humidity of 60–75%). The ‘Orin’ calli were subcultured in culture dishes (9 cm in diameter) containing 35 mL of Murashige and Skoog (MS) medium (0.4 mg L^−1^ 6-BA, 1.5 mg L^−1^ 2,4-D, 30 g L^−1^ sucrose and 7.5 g L^−1^ agar; pH 5.8–6.0; autoclaved at 121 °C for 20 min). Tissue-cultured plants of the apple cultivar ‘Gala’ were incubated under greenhouse conditions of 24 ± 0.5 °C and a 16-h light/8-h dark cycle (at a relative humidity of 60–75%). The ‘Gala’ explants were cultured in culture bottles (5.5 cm in diameter) containing 40 mL of MS subculture medium (0.5 mg L^−1^ 6-BA, 0.2 mg L^−1^ IAA, 30 g L^−1^ sucrose and 7.5 g L^−1^ agar; pH 5.8–6.0; autoclaved at 121 °C for 20 min). *Botryosphaeria dothidea* was incubated in culture dishes (9 cm in diameter) containing 15 mL of potato dextrose agar medium at 24 ± 0.5 °C in darkness. *Nicotiana benthamiana* seeds were surface-sterilised and germinated on MS medium under greenhouse conditions of 24 ± 0.5 °C and a 16-h light/8-h dark cycle (at a relative humidity of 60–75%). *N*. *benthamiana* seedlings were subsequently transplanted at the two-leaf or three-leaf stage into soil and grown under greenhouse conditions.

### Vector construction and genetic transformation

Fragments and full-length coding sequences were cloned from a cDNA library that was reverse-transcribed from RNA isolated from Fuji fruits synthesised using a First Strand cDNA Synthesis Kit (Thermo Fisher Scientific, USA) in accordance with the manufacturer’s instructions. PCR was performed with Pfu/Taq polymerase (Fermentas, USA). The primers used are shown in Supplementary Table S1. All generated amplicons were subcloned into a pLB vector (Tiangen, China). Homologous MdSYP121 protein sequences were retrieved from the National Center Biotechnology Information (NCBI) database and aligned using DNAMAN 5.2.2 software (Lynnon Biosoft, USA). A phylogenetic tree was subsequently generated using the Neighbour Joining method with MEGA 5.0 software. A *MdSYP121* fragment from 121 to 613 bp was used for RNAi vector construction. The *MdSYP121* RNAi fragments were subsequently transferred to pHANNIBAL and pCB302 vectors by T4 recombination (NEB, USA) to generate RNAi transformation vectors. The full-length products were used to create HA protein fusions by T4 recombination into pCB302-MdSYP121-HA under the control of the cauliflower mosaic virus (CaMV) 35S promoter to generate the overexpression (OE) transformation factor.

The leaves of 4-week-old tissue-cultured ‘Gala’ plants were transformed with *Agrobacterium tumefaciens* LBA4404 carrying the RNAi transformation vector. The leaves were scratched and pre-differentiated on MS differentiation medium (2.0 mg L^−1^ TDZ, 0.2 mg L^−1^ IAA, 30 g L^−1^ sucrose and 7.5 g L^−1^ agar; pH 5.8–6.0; autoclaved at 121 °C for 20 min) for 2 days, and then they were incubated with LBA4404 (optical density (OD) = 0.4–0.6) for 20–30 min and co-cultured on MS differentiation medium at 24 °C for 7 days in the dark. The leaves were then transferred to screening differentiation medium containing glufosinate ammonium and carbenicillin under a 16-h light/8-h dark cycle (at a relative humidity of 60–75%). The screened explants were cultured on MS subculture medium.

To generate transgenic apple calli, 10-day-old apple calli were transformed with *A. tumefaciens* LBA4404 carrying the RNAi or OE transformation vectors, respectively. The apple calli were incubated with LBA4404 (optical density (OD) = 0.4–0.6) for 20–30 min and co-cultured on MS solid media containing no antibiotics at 24 °C for 48 h in the dark. The calli cells were then transferred to screening medium containing glufosinate ammonium and carbenicillin^22^.

### Subcellular localisation of MdSYP121 and MdSNAP33

The open reading frames (ORFs) of *MdSYP121* and *MdSNAP33* were inserted into a pCB302 green fluorescent protein (GFP) vector, whose N-terminus consists of a GFP under the control of the CaMV 35S promoter. To achieve transient expression, the recombinant plasmids pCB302-MdSYP121-GFP and pCB302-MdSNAP33-GFP were transformed into *A. tumefaciens* GV3101. After the cell cultures were incubated overnight, *A. tumefaciens* cells were harvested via centrifugation and resuspended in infiltration medium (100 mL of medium containing 1 mL of 1 M MES-KOH at pH 5.6, 333 μL of 3 M MgCl_2_ and 100 μL of 150 mM acetosyringone). The leaves of 5-week-old *N. benthamiana* plants were used for transient expression, and the GFP signals were observed using a LSM 880 META confocal microscope (Carl Zeiss, Germany). A pCB302-GFP construct was used as a control.

### RNA extraction and quantitative real-time PCR (qRT-PCR) analysis

Total RNA was isolated from apple tissue culture plants and calli using the cetyltrimethylammonium bromide method described by Wang et al.^23^. First strand cDNA was synthesised using a First Strand cDNA Synthesis Kit (Thermo Fisher Scientific, USA) in accordance with the manufacturer’s instructions. qRT-PCR was used to detect the expression levels of the target genes. The 20 μL PCR mixture comprised 10 μL of Fast Start Universal SYBR^®^ Green Master mix (Roche, USA), 0.6 μL of each primer (10 mM), 2 μL of diluted cDNA and 6.8 μL of PCR-grade H_2_O and a CFX96^TM^ Real-time Detection System (Bio-Rad, USA) was used to perform PCR. The following PCR programme was used: predenaturation at 98 °C for 10 min; 40 cycles of 98 °C for 15 s and then 60 °C for 30 s; and a final melt cycle from 60 to 98 °C. The PCR process was completed with a melting curve analysis program. The *Malus* × *domestica actin* gene was used as a standard control to quantify cDNA abundance. The primers used in the qRT-PCR analyses are shown in Supplementary Table S2.

### Pathogen infection assays

For the pathogen infection analysis, transgenic and Vec (serving as an empty vector control) lines were transferred to MS solid medium that lacked glufosinate ammonium and carbenicillin. Ten-day-old Vec, RNAi and OE lines were infected with 0.5-cm-diameter agar discs containing uniform *B. dothidea* mycelia that were incubated for 5 days. The calli were co-cultured for 4 days in the dark. For expression pattern analyses, treated calli were collected from culture dishes, frozen in liquid nitrogen and stored at −80 °C for RNA extraction. Each experimental treatment was repeated at least three times.

### Enzyme activity assays

The activity levels of peroxidase (POD), catalase (CAT), ascorbate peroxidase (APX) and glutathione reductase (GST) were spectrophotometrically measured using hydrogen peroxide test kits (Nanjing Jiancheng Bioengineering Institute, China). Each experimental treatment was repeated at least three times.

### Library construction and RNA-Seq analysis

Nine independent calli from RNAi and Vec lines were infected by *B. dothidea*. Both the total composite RNA from the RNAi lines and the total composite RNA from the Vec lines, respectively, were used for Illumina sequencing at Novogene Technologies (Beijing, China). All procedures during the cDNA library construction were performed in accordance with a standard Illumina sample preparation protocol. The RNA-Seq libraries were sequenced on an Illumina Genome HiSeq platform.

After the RNA was sequenced, the adaptors were trimmed and low-quality sequences were removed from the raw data. The unigenes were annotated using the following databases: the Clusters of Orthologous Groups (COG) (http://www.ncbi.nlm.nih.gov), Gene Ontology (GO) (http://www.geneontol-ogy.org/) and Kyoto Encyclopaedia of Genes and Genomes (KEGG) (http://www.genome.jp/kegg/)^24–26^. To assay the differentially expressed genes (DEGs), trimmed mean of M-values normalisation and DEG seq were used to normalise gene expression levels for differential expression analyses, respectively^27^. A *q*-value < 0.005 served as the *P*-value threshold in multiple tests to determine the significance of differences in gene expression.

### Verification of RNA-Seq data using qRT-PCR

RNA was extracted from each RNAi and Vec line to verify the RNA-Seq data. RNA was prepared, and cDNA was synthesised, respectively, as described above. The primers used for the qRT-PCR analyses are shown in Supplementary Table S2. All of the samples were tested at least three times, and the experiments were performed on three biological replicates.

### GST pull-down analysis

MdSYP121 with an HA tag was expressed in apple calli. The calli were homogenized in an extraction buffer containing 50 mM Tris-HCl (pH 7.5), 150 mM NaCl, 50 mM EDTA, 1% Triton, 1 mM phenylmethanesulfonyl fluoride and a protease inhibitor cocktail (Roche, USA). For prokaryotic expression assays, a plasmid containing MdSNAP33 with a GST tag was transformed into *Escherichia coli* Rosetta (Tiangen, China), and the proteins were induced with 1 mM isopropyl 1-thio-*β*-d-galactopyranoside at 28 °C. The GST-MdSNAP33 protein was purified using a Pierce^®^ GST Spin Purification Kit in accordance with the manufacturer’s protocol (Thermo Fisher Scientific, USA). The mixture of MdSYP121-HA protein extracted from the apple calli and the purified GST-MdSNAP33 protein with 2 μL of GST antibody were incubated together with gentle shaking for 2 h at 4 °C. The protein mixture was then immunoprecipitated with G-agarose beads (Sigma-Aldrich, USA) by gentle shaking for 2 h at 4 °C. The beads were collected and washed three times with washing buffer (100 mM NaCl, 10 mM HEPES, pH 7.5; 1 mM EDTA; a protease inhibitor cocktail; and 10% glycerol) and once with 50 mM Tris-HCl (pH 7.5). The immunoprecipitated proteins were analysed with an HA antibody. Equal amounts of total protein were electrophoresed on 10% SDS-PAGE.

### Bimolecular fluorescence complementation assays

Full-length *MdSYP121* and *MdSNAP33* were fused to yellow fluorescent protein (YFP) vectors to pUC-SPYCE-35S and pUC-SPYNE-35S, respectively, by T4 recombination. The primers used are listed in Supplementary Table S1. These two recombinant plasmids were transiently expressed in tobacco leaves by *A. tumefaciens* (GV3101)-mediated infiltration^28^. The YFP fluorescence of tobacco leaves was detected after infiltration for 4 days using a LSM 880 META confocal microscope (Carl Zeiss, Germany).

### SDS resistance assays

For in vitro-binding studies, MdSYP121-HA homogenized in extraction buffer (as mentioned above) and GST-MdSNAP33 purified protein (as mentioned above) were incubated together for 12 h at 4 °C (head-over-head rotation). The bound material was eluted by incubation (30 min at 37 °C) together with 4× SDS loading buffer, because it is SDS-resistant but heat-sensitive; the controls were incubated for 10 min at 100 °C^29^. Protein samples were then separated on 10% SDS-PAGE. Western blot was used to detect the expression of proteins.

### Statistical analysis

All experiments were performed at least three times. The error bars in each graph indicate the mean values ± SEs of three repetitions. Statistical significance between different measurements was determined using Tukey’s honestly significant difference (HSD) test via IBM Statistical Product and Service Solutions (SPSS) statistics software version 19 (IBM, USA).

## Results

### Cloning and characterisation of MdSYP121

To study the resistance mechanism of apple to apple ring rot, we analysed the RNA-Seq data of Fuji cultivars inoculated with *B. dothidea*. MDP0000709455 was chosen for detailed characterisation because it was induced in ‘Fuji’ apple with *B. dothidea* inoculation and served as an important node by analysing the interaction network using Cytoscape (version 3.6.0, USA) (data not shown), and the orthologous genes of MDP0000709455 played critical roles in controlling disease resistance towards powdery mildew in Arabidopsis and barley^10^. A fragment was isolated from the cDNA library of Fuji fruit using the primers listed in supplementary Table S1. The full-length cDNA sequence consisted of a 1068-bp ORF. The ORF encoded a protein that was 356 amino acids in length and had a calculated molecular mass of 43.83 kDa and an isoelectric point of 7.15. This clone exhibited a high level of sequence similarity to AtSYP121 of Arabidopsis. Based on multiple sequence alignments with other plant SYP121s, MDP0000709455 contains Ha-conserved, Hb-conserved and Hc-conserved subdomains, a Qa-SNARE subdomain and a transmembrane domain (Supplementary Fig. S1a). In addition, MDP0000709455 exhibits high homology to AtSYP121 (AEE75103), PpSYP121 (XP_007202232), CsSYP121 (XP_00648162) and OsSYP121 (ABB22872) (Supplementary Fig. S1a); so, this gene was designated *MdSYP121*.

To investigate the evolutionary relationships among SYP121s from different species, a phylogenetic analysis based on their amino acid sequences was performed by the Neighbour Joining method using the software MEGA version 5.0. As shown in Supplementary Fig. S1b, MdSYP121 exhibited high similarity to the members of the Qa-SNARE group, including AtSYP121, PpSYP121, CsSYP121 and QsSYP121. These results suggest that MdSYP121 is a member of the Qa-SNARE.

### Subcellular localisation of MdSYP121

**Fig. 1:**
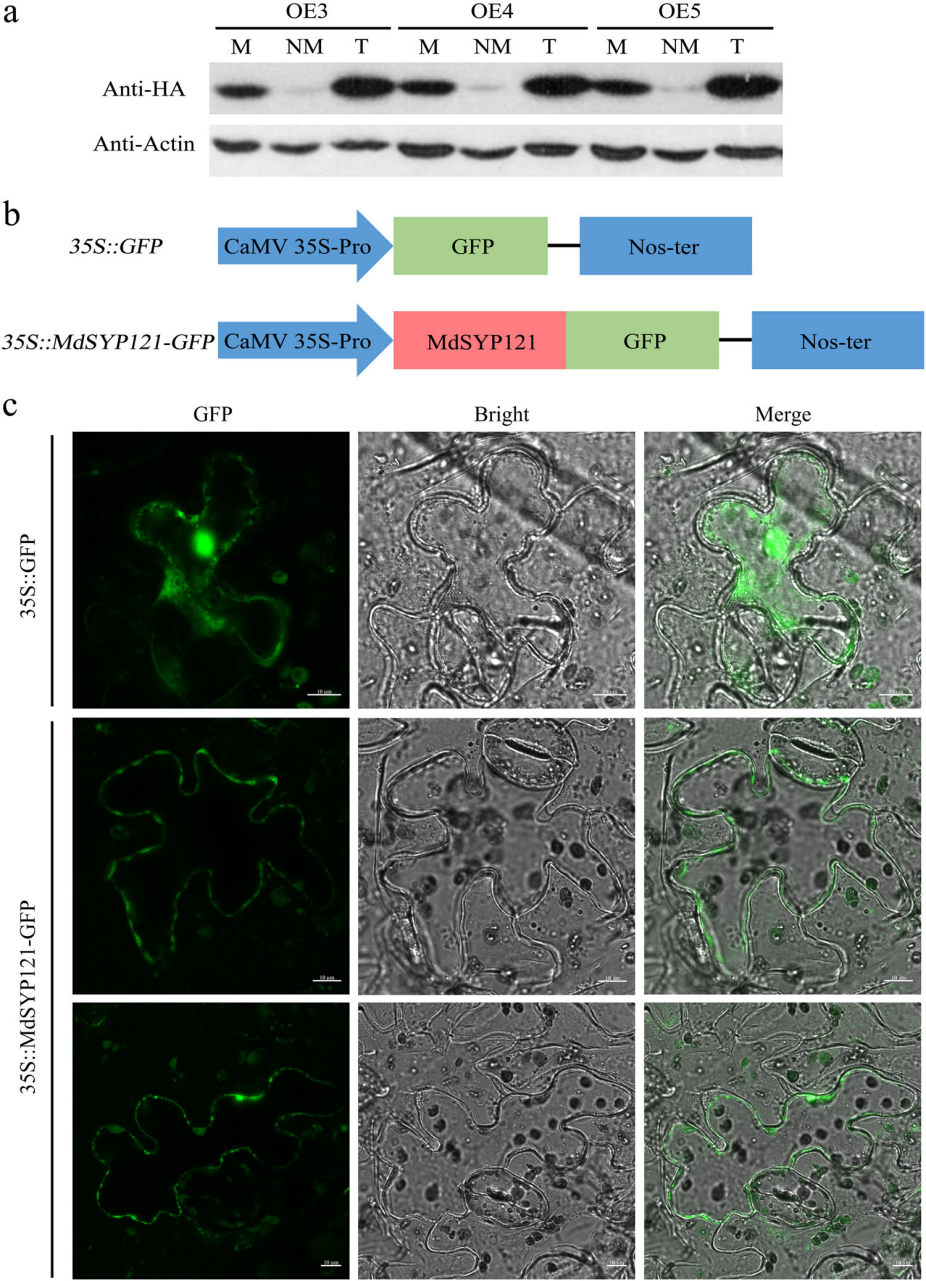
Subcellular localisation of the MdSYP121 protein in apple calli and *N. benthamiana*. **a** Expression of 35S::MdSYP121-HA fusion constructs in apple calli. Total proteins, non-membrane proteins and membrane proteins (indicated in the figure as T, NM and M, respectively) were detected using anti-HA. **b** Schematic diagram of the 35S::MdSYP121-GFP fusion construct and the 35S::GFP construct. **c** Transient expression of the 35S::MdSYP121-GFP fusion construct and the 35S::GFP construct in *N. benthamiana*. Green fluorescence was observed after transient infection for 4 days using a confocal microscope. Bar = 10 μm

To determine the actual localisation of the MdSYP121 protein within cells, MdSYP121 was fused to an HA tag in the OE vector 35S::MdSYP121-HA; the recombinant plasmid was transferred into apple calli, and OE transgenic lines were obtained (Supplementary Fig. S2). The membrane proteins of the MdSYP121-OE lines were separated using a membrane protein extraction kit (BestBio, China). MdSYP121 was detected among both total proteins and membrane proteins by using western blot with anti-HA antibody (Fig. 1a).

In addition, MdSYP121 was fused to a GFP tag in the OE vector 35S::MdSYP121-GFP (Fig. 1b). The fusion protein was transiently expressed in *N. benthamiana* leaves using agroinfiltration. A 35S::GFP construct served as a control. Fluorescence was detected in the PM of *N. benthamiana* leaves (Fig. 1c). These results suggested that MdSYP121 is a membrane-localised protein.

### Silencing MdSYP121 increased resistance to *Botryosphaeria dothidea*

To investigate the function of *MdSYP121*, we silenced *MdSYP121* in apple ‘Gala’ using the RNAi method. However, the transgenic plants showed the dwarfism and necrosis phenotypes, and only two transgenic plants were ultimately obtained (Supplementary Fig. S3). The transgenic plants were tiny and difficult to differentiate, so the functional analysis was difficult to perform.

To further analyse the function of this gene, we produced calli tissue whose expression of *MdSYP121* was inhibited by the RNAi method. Ten independent transgenic lines were selected using glufosinate ammonium resistance selection and qRT-PCR (Supplementary Fig. S4). Three typical MdSYP121-RNAi lines (RNAi3, RNAi5 and RNAi7) were randomly selected for further functional analysis. As a control, Vec lines were subcultured at the same time.

**Fig. 2:**
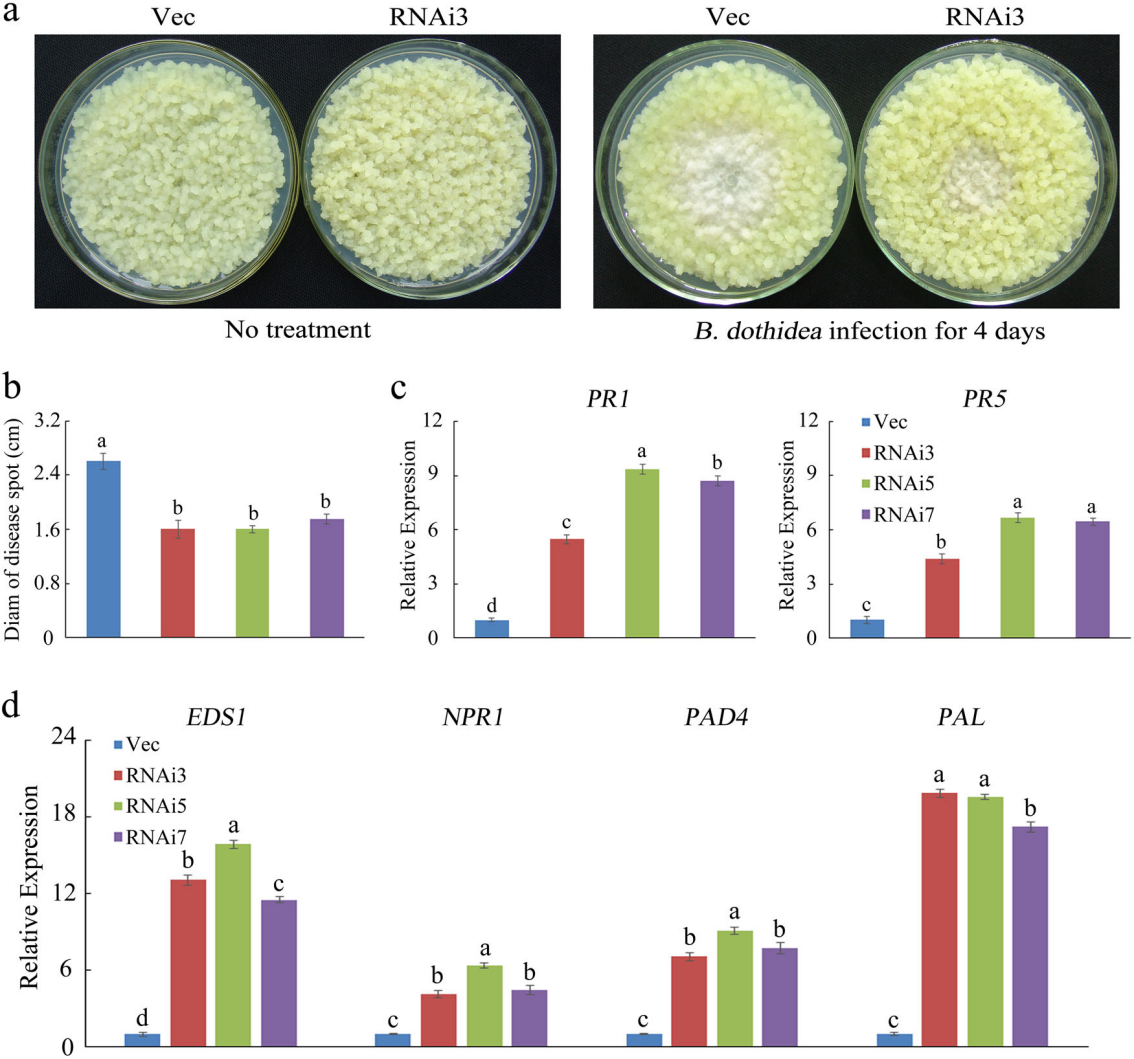
MdSYP121-RNAi calli lines enhance resistance to *B. dothidea*. **a** Representative phenotypes of Vec and MdSYP121-RNAi calli both untreated and infected with *B. dothidea* for 4 days, respectively. **b** Pathogen disease indexes in Vec and MdSYP121-RNAi calli lines after *B. dothidea* infection for 4 days. **c** qRT-PCR analysis for the expression of SA signalling-related genes in Vec, RNAi3, RNAi5 and RNAi7 calli lines after *B. dothidea* infection for 4 days. **d** qRT-PCR analysis for the expression of SA-related genes in Vec, RNAi3, RNAi5 and RNAi7 calli lines after *B. dothidea* infection for 4 days. The error bars in **b**, **c** and **d** indicate the mean values ± SEs of three independent experiments (*n* = 6). The letters above the columns represent significant differences (*P* < 0.05) based on Tukey’s HSD test. Vec served as the empty vector control

To identify the function of *MdSYP121* during *B. dothidea* infection, MdSYP121-RNAi lines growing on MS agar medium were inoculated with *B. dothidea*. Although the untreated Vec and MdSYP121-RNAi lines have slight difference, the spot extension areas of the MdSYP121-RNAi lines were clearly fewer than those of the Vec lines after inoculation with *B. dothidea* for 4 days (Fig. 2a and Supplementary Fig. S5b). The RNAi lines showed a nearly twofold decrease in fungal growth (Fig. 2b).

The phytohormone SA serves as an endogenous messenger during biotic stress in plants and is required for the activation of systemic acquired resistance (SAR)^30^. The expression of SA signalling-related and synthesis-related genes was analysed in the MdSYP121-RNAi and Vec lines after inoculation with *B. dothidea* for 4 days. As shown in Fig. 2c,d, the expression levels of both the SA signalling-related genes *PR1*, *PR5* and *NPR1* and the SA synthesis-related genes *EDS1*, *PAD4* and *PAL* were higher in the MdSYP121-RNAi lines than those in the Vec line. These results show that silencing *MdSYP121* increases tolerance to *B. dothidea*.

### Overexpression of MdSYP121 decreased resistance to *Botryosphaeria dothidea*

To investigate the biological role of *MdSYP121*, we produced calli tissue that overexpressed *MdSYP121*. Six independent transgenic lines were selected using glufosinate ammonium resistance selection and western blot analysis (Supplementary Fig. S2). Three typical lines (OE3, OE4 and OE5) were randomly selected and confirmed, and these lines were used for further functional analysis. As a control, Vec lines were subcultured at the same time.

**Fig. 3:**
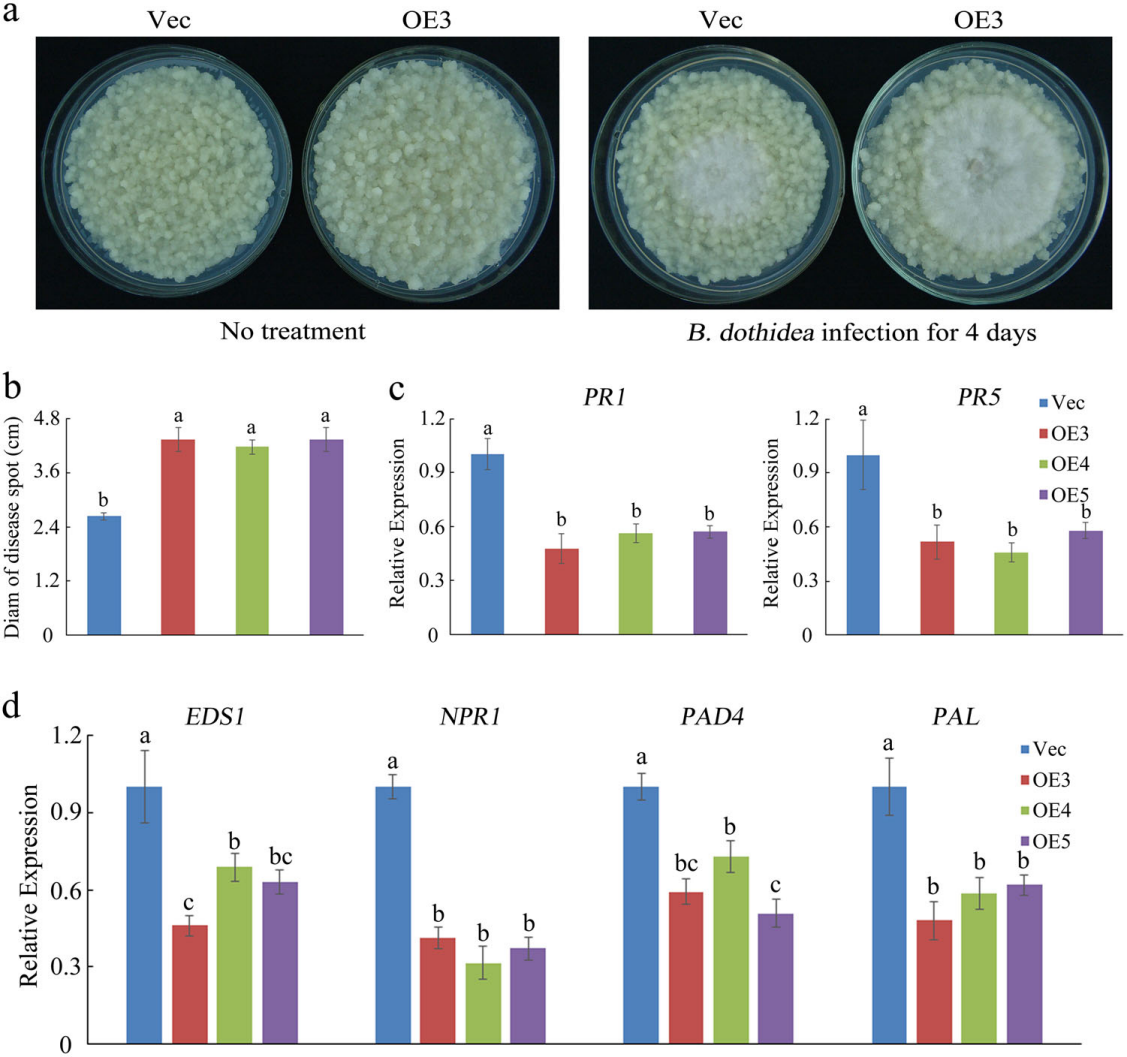
MdSYP121-OE calli lines reduce resistance to *B. dothidea*. **a** Representative phenotypes of Vec and MdSYP121-OE calli lines both untreated and infected with *B. dothidea* for 4 days, respectively. **b** Pathogen disease indexes in Vec and MdSYP121-OE calli lines after *B. dothidea* infection for 4 days. **c** qRT-PCR analysis for the expression of SA signalling-related genes in Vec, OE3, OE4 and OE5 calli lines after *B. dothidea* infection for 4 days. **d** qRT-PCR analysis for the expression of SA-related genes in Vec, OE3, OE4 and OE5 calli lines after *B. dothidea* infection for 4 days. The error bars in **b**, **c** and **d** indicate the mean values ± SEs of three independent experiments (*n* = 6). The letters above the columns represent significant differences (*P* < 0.05) based on Tukey’s HSD test. Vec served as the empty vector control

As shown in Fig. 3a and Supplementary Fig. S5a, 4 days after *B. dothidea* inoculation, fungal extension was significantly greater in the OE lines than that in the Vec lines. Compared with that of the Vec lines, the spot extension areas of the OE lines exhibited a nearly twofold increase in fungal growth (Fig. 3b).

The SA signalling-related and synthesis-related genes were also analysed in the MdSYP121-OE and Vec lines after *B. dothidea* infection for 4 days. As shown in Fig. 3c,d, the expression of the SA-related genes was lower in the MdSYP121-OE lines than that in the Vec lines. These results show that overexpressing *MdSYP121* decreased tolerance to *B. dothidea*.

### Silencing *MdSYP121* influenced the oxidative activity of plants

**Fig. 4:**
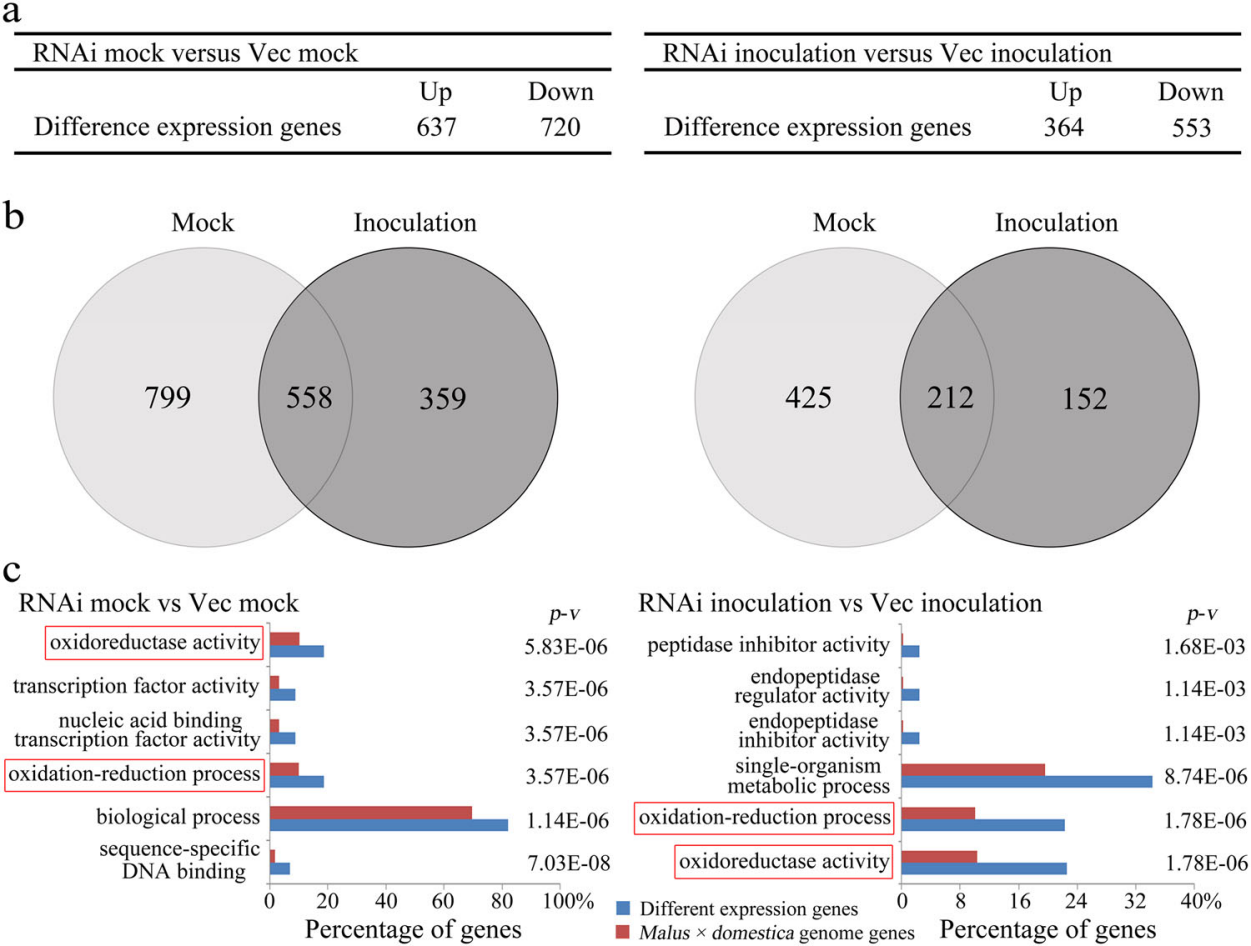
Transcriptome analysis of Vec and MdSYP121-RNAi calli lines. **a** The number of DEGs (twofold change cut-off) between Vec and MdSYP121-RNAi calli lines under mock conditions or in response to *B. dothidea* infection. **b** Venn diagram showing the numbers of genes whose expression was independent or dependent of *B. dothidea* infection. Left: the expression of genes regulated with or without *B. dothidea* inoculation. Right: the expression of genes upregulated with or without *B. dothidea* inoculation. Mock indicates the RNAi mock compared with the Vec mock; inoculation indicates RNAi with *B. dothidea* inoculation compared with Vec with *B. dothidea* inoculation. **c** GO analysis of DEGs independent or dependent of *B. dothidea* infection. Histograms of the values were generated to highlight the GO enrichment of representative GO classes. *P*-values for each enriched class are indicated (*p*–*v*)

To further analyse the molecular mechanism of *MdSYP121* involved in *B. dothidea* resistance, we performed an RNA-Seq analysis of Vec and MdSYP121-RNAi calli lines under mock conditions or 4 days after treatment with *B. dothidea*. After trimming the adaptor sequences and removing low-quality reads, we generated 29.65 Gb of clean reads. Among these unigenes, many DEGs were identified between the Vec and RNAi samples: in the absence of *B. dothidea* inoculation, 637 genes were upregulated in the RNAi samples, and 720 genes were downregulated; after *B. dothidea* inoculation, 364 genes were upregulated and 553 genes were downregulated (>twofold changes) (Fig. 4a,b).

**Fig. 5:**
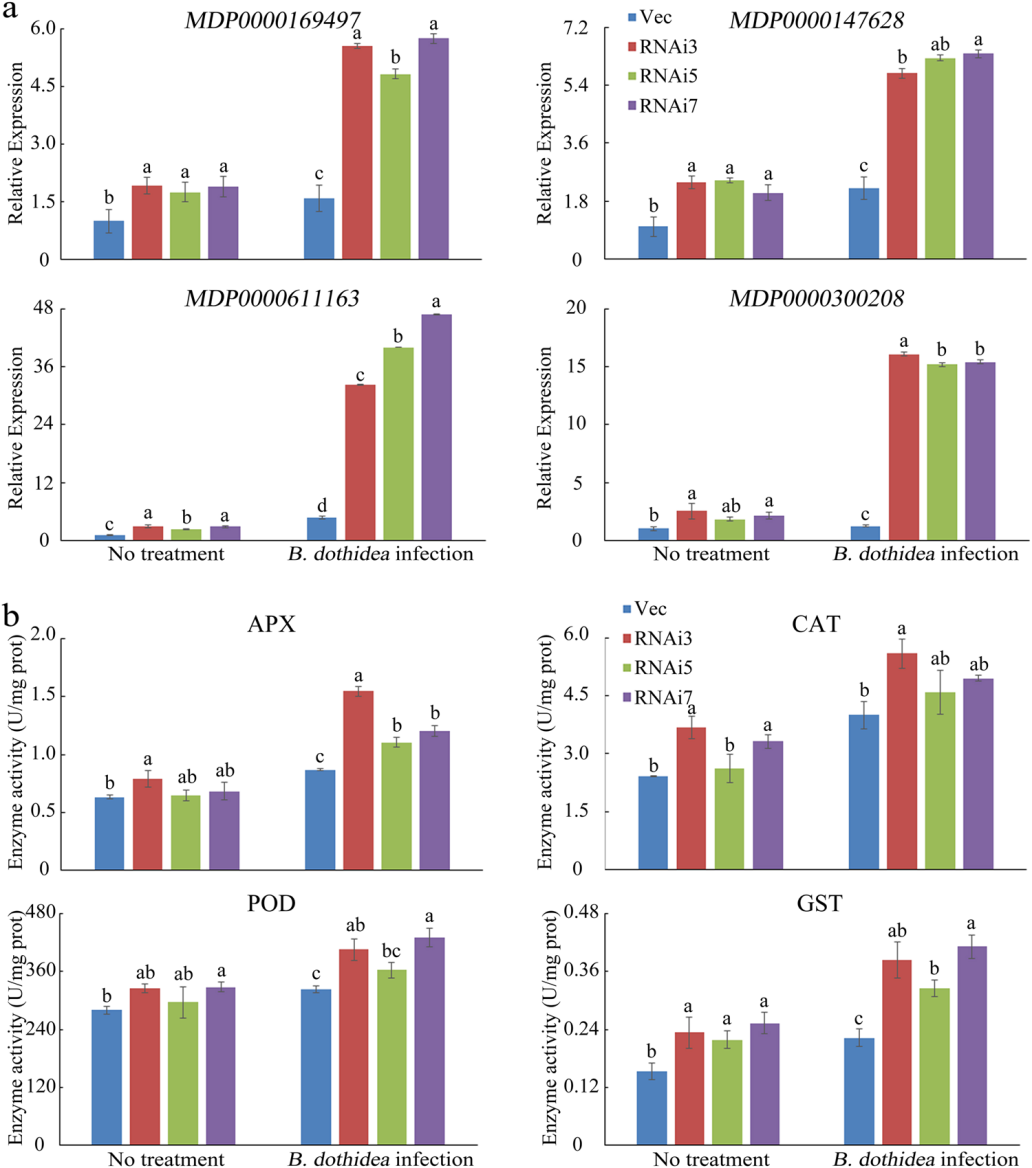
Expression of antioxidant enzyme genes and analysis of the activities of the antioxidant enzymes in Vec and MdSYP121-RNAi calli lines. **a** qRT-PCR analysis of four genes randomly selected from transcriptomic experiments following inoculation with *B. dothidea* for 4 days and untreated with *B. dothidea* in the Vec and MdSYP121-RNAi calli lines. **b** The activities of the antioxidant enzymes in Vec and RNAi calli lines after inoculation with *B. dothidea* for 4 days and in untreated controls. The error bars in **a** and **b** indicate the mean values ± SEs of three independent experiments (*n* = 6). The letters above the columns represent significant differences (*P* < 0.05) based on Tukey’s HSD test

To analyse functional differences between the Vec and MdSYP121-RNAi samples, the identified DEGs were characterised using GO enrichment analysis to explore their relevant biological functions. Based on the GO annotation analysis of these genes, we found that the oxidation–reduction process (GO:0055114) and oxidoreductase activity (GO:0016491) were the major enriched GO terms among the upregulated genes (Fig. 4c and Table 1). Oxidation–reduction enzymes have important functions; they can function as part of protective mechanisms involved in plant disease resistance. To test whether the *MdSYP121* is related to oxidative regulation, four genes from GO:0055114 or GO:0016491 were randomly selected and verified using qRT-PCR. We confirmed that the expression of the selected genes in the MdSYP121-RNAi lines (RNAi3, RNAi5 and RNAi7) showed the same increasing tendency and that the expression was higher in those lines than in the Vec lines (Fig. 5a). We then analysed the related oxidation–reduction enzyme activity in the Vec and MdSYP121-RNAi lines. As shown in Fig. 5b, the activity of oxidation–reduction enzymes was higher in the MdSYP121-RNAi transgenic lines than that in the Vec lines both in the presence and absence of *B. dothidea* infection. In conclusion, these results show that *MdSYP121* plays a vital role in plant disease resistance together with a set of reactive oxygen biotic stress genes.

**Table 1:** Portion of upregulated stress-related genes involved in oxidoreductase, oxidation–reduction, single-organism metabolism and biological processes and the response to stress in response to *B. dothidea*

Gene ID	Annotation	RNAi mock vs Vec mock (log2 fold-change)	*p*–*v*	RNAi inocation vs Vec inocation (log2 fold-change)	*p*–*v*
Oxidoreductase activity					
MDP0000735747	Gibberellin 2-beta-dioxygenase 2	1.5984	4.49E−14	1.6161	6.00E−09
MDP0000136847	(R)-mandelonitrile lyase 2	1.4548	2.48E−05	2.2236	3.58E−79
MDP0000158739	Cytochrome P450	2.4089	2.36E−24	3.4183	6.91E−129
MDP0000742438	Ferric reduction oxidase 4	2.2491	4.77E−67	2.3085	9.05E−90
MDP0000570102	Protein SRG1	1.7835	3.81E−62	1.8106	1.22E−78
MDP0000611163	Peroxidase	—	—	2.3526	8.70E−16
MDP0000925883	UDP-glucuronic acid decarboxylase 5	1.4681	3.87E−19	1.694	7.17E−29
MDP0000451182	Peroxidase 66	—	—	4.8509	1.20E−08
MDP0000251295	1-aminocyclopropane-1-carboxylate oxidase	—	—	1.652	3.36E−24
MDP0000234983	Cytokinin dehydrogenase 5	—	—	1.6208	0.00037497
MDP0000442206	Glutamate synthase [NADH]	—	—	2.3915	1.18E−51
MDP0000306998	Tropinone reductase	—	—	1.0335	1.95E−05
Oxidation–reduction process					
MDP0000509613	NAD(P)H dehydrogenase	1.2347	4.67E−33	1.6215	0
MDP0000255970	Transcription factor DIVARICATA	1.0258	0.0011272	1.1359	0.00014298
MDP0000555589	Polyphenol oxidase	4.0991	8.44E−38	4.0455	1.55E−40
MDP0000286750	Ferritin-3, chloroplastic	—	—	4.2438	0.00059104
MDP0000807470	Sorbitol dehydrogenase	—	—	1.0825	5.59E−07
MDP0000312559	Probable nucleoredoxin 2	—	—	2.3805	0.00037441
MDP0000906067	Photosystem II repair protein	—	—	2.51	3.21E−09
MDP0000735022	Beta-glucosidase 24	—	—	1.8177	6.52E−12
MDP0000130200	Primary amine oxidase	—	—	1.5744	4.77E−05
Single-organism metabolic process					
MDP0000287919	4-hydroxycoumarin synthase 1	2.105	1.73E−16	2.5539	0
MDP0000257119	4-hydroxycoumarin synthase 2	—	—	3.8812	0.00059684
MDP0000180326	Methionine gamma-lyase	4.8137	5.89E−10	5.1866	2.42E−28
MDP0000266097	Dephospho-CoA kinase	1.0462	4.42E−37	1.3943	3.29E−120
MDP0000289339	Cellulose synthase-like protein	1.9853	1.93E−128	2.21	9.22E−192
MDP0000180890	Probable aminotransferase	2.3741	4.80E−05	4.265	2.66E−19
MDP0000319502	Bark storage protein	—	—	1.1989	1.68E−12
MDP0000239026	Ocs element-binding factor 1	—	—	1.8719	1.25E−06
MDP0000219975	Acetyl-CoA carboxylase 1	—	—	1.9818	2.98E−35
MDP0000133520	Patatin-like protein 2	—	—	1.9469	3.35E−18
MDP0000137919	CTP synthase	—	—	2.7395	3.58E−06
MDP0000135529	Alpha-amylase	—	—	2.654	7.22E−05
Biological process					
MDP0000280265	Acidic endochitinase	1.4679	1.3334E−38	1.9457	0
MDP0000390049	Tryptophan aminotransferase-related protein 4	1.4662	1.2133E−47	1.7021	2.72E−245
MDP0000199977	SPX domain-containing membrane protein	1.2495	0.0049554	1.5426	2.9762E−07
MDP0000287302	Thaumatin-like protein	1.2316	2.79E−150	1.6295	0
MDP0000180890	Probable aminotransferase	2.3741	4.7997E−05	4.265	2.657E−19
MDP0000321210	50S ribosomal protein L18	1.8452	2.2985E−05	2.564	1.4141E−08
MDP0000126058	Rhodanese-like domain-containing protein 4	1.1419	3.6208E−08	1.3563	3.8163E−11
MDP0000179851	Caffeic acid 3-O-methyltransferase 1	—	—	1.7312	0.00075589
MDP0000230504	Probable aldo-keto reductase 1	—	—	1.068	2.7665E−11
MDP0000321125	Late embryogenesis abundant protein 2	—	—	5.3001	0.0032804
MDP0000805281	Cyanogenic beta-glucosidase	—	—	2.5017	1.287E−191
MDP0000490846	Uncharacterised protein Mb2734	—	—	1.0332	8.7225E−06
MDP0000937817	Calcineurin B-like protein 4	—	—	1.0809	4.7002E−06
MDP0000128326	Ethylene-responsive transcription factor	—	—	3.1451	0.0022472
Response to stress					
MDP0000782085	Pathogenesis-related protein	1.1335	2.169E−177	1.1759	0
MDP0000642609	Universal stress protein (USP) family protein	2.3681	1.8358E−20	2.8868	1.7317E−89
MDP0000846849	Glu *S.griseus* protease inhibitor	2.5176	8.5997E−37	2.8343	0
MDP0000471879	Proteinase inhibitor	1.2793	6.0407E−09	2.0045	3.522E−151
MDP0000313454	Major allergen Pru ar 1	4.8158	0.00032033	5.4776	5.4005E−16
MDP0000265782	Heat shock protein 90	—	—	1.0131	7.2559E−07
MDP0000327191	Glucan endo-1,3-beta-glucosidase	—	—	4.417	0.0013841
MDP0000868045	Dehydrin DHN1	—	—	1.6711	5.857E−301

*P*-values for each enriched class are indicated (*p*–*v*)

### MdSYP121 interacted with MdSNAP33

**Fig. 6:**
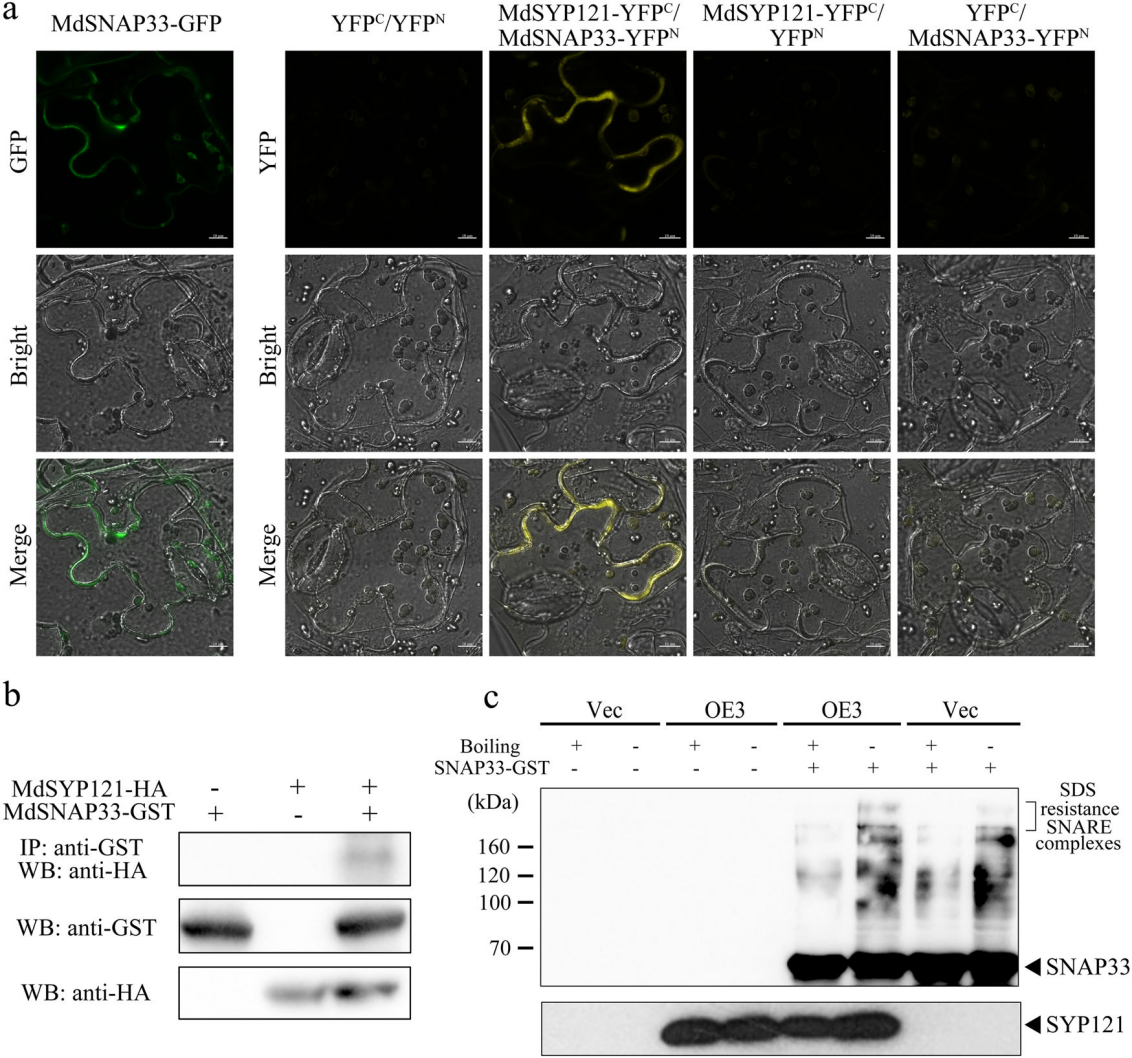
MdSYP121 interacts with MdSNAP33 and forms a SNARE complex. **a** Transient expression of a 35S::MdSNAP33-GFP fusion construct in *N. benthamiana*. Green fluorescence was observed after transient infection for 4 days using a confocal microscope. Bars = 10 μm. In vivo interactions between MdSYP121 and MdSNAP33 were determined using BiFC. C-terminal and N-terminal fragments of YFP (indicated in the figure as YFP^C^ and YFP^N^, respectively) were fused to the C-terminus of MdSYP121 and MdSNAP33, respectively. Combinations of YFP^N^ and YFP^C^ with the corresponding MdSYP121 and MdSNAP33 constructs were used as negative controls. Fluorescence of YFP represents protein-protein interactions. Yellow fluorescence was observed using a confocal microscope after transient infection for 4 days. Bars = 10 μm. **b** GST pull-down assays were performed using HA antibody. Western blot analysis results showing protein expression levels. **c** MdSYP121 together with MdSNAP33 forms SDS-resistant SNARE complexes. SDS-resistant MdSYP121-containing complexes are detectable in Vec and MdSYP121-OE calli lines. Proteins were extracted from Vec and MdSYP121-OE lines and incubated together with GST-MdSNAP33 protein. The proteins were loaded on a 10% polyacrylamide gel and subjected to immunoblot analysis in conjunction with anti-GST. The lower panel shows the same immunoblot protein samples that were detected with anti-HA

Binary target membrane (t)-SNARE complexes are typically formed by SYP121 and SNAP25 at the PM during exocytosis. The Arabidopsis *SNAP25* homologue, *SNAP33*, has been detected in the target tissue of powdery mildew fungi in leaves^12^. In the present study, the full length of *MdSNAP33* (MDP0000242070) was cloned and analysed in apple. In Arabidopsis, AtSNAP33 is located in the PM^3,31,32^. To determine the actual localisation of the protein within cells, MdSNAP33 was fused to a GFP tag in the OE vector 35S::MdSNAP33-GFP. The fusion protein was transiently expressed in *N. benthamiana* leaves using agroinfiltration. The result showed that fluorescence was detected in the PM in *N. benthamiana* leaves (Fig. 6a).

Interaction between MdSYP121 and MdSNAP33 was verified by bimolecular fluorescence complementation (BiFC) assays using tobacco leaves. Co-expression of MdSYP121-YFP^C^ and MdSNAP33-YFP^N^ produced strong signals in the PM (Fig. 6a). As negative combinations, YFP^N^/MdSYP121-YFP^C^, MdSNAP33-YFP^N^/YFPC and YFP^N^/YFP^C^ produced no detectable fluorescence signals.

The physical interaction between MdSYP121 and MdSNAP33 was also examined by pull-down analysis. We performed MdSYP121 pull-down assays using an antibody against the HA epitope, and the presence of MdSNAP33 was assayed using an antibody against the GST epitope (Fig. 6b). Immunoblot analysis with an anti-HA antibody indicated that MdSYP121 co-immunoprecipitated by MdSNAP33. These findings indicated that MdSYP121 specifically interacts with MdSNAP33.

SYP121 forms SNARE complexes with SNAP33 adaptors and two functionally redundant VAMP72 subfamily members, VAMP721/VAMP722; these complexes are important for defence resistance in Arabidopsis^12^. SYP121-dependent disease resistance acts in vivo mainly via SNAP33 and VAMP721/VAMP722^12^. The typical features of exocytic ternary SNARE complexes are SDS resistance and heat sensitivity in animals and yeast^29^. We detected SDS-resistant SNARE complexes by incubating MdSYP121-HA protein extracted from apple calli together with GST-MdSNAP33 protein bound to glutathione sepharose. Sedimented bead-bound GST-MdSNAP33 and MdSYP121-HA were released and detected by immunoblot analysis using GST and HA antibodies, respectively (Fig. 6c). The GST-MdSNAP33 protein and MdSYP121-HA apple calli proteins were able to form SDS-resistant and heat-sensitive complexes. These results showed that the MdSYP121 can form a SNARE complex together with MdSNAP33.

## Discussion

Many studies have reported that SYP121 is an important element in host and/or non-host resistance, and SYP121 may play a role in penetration resistance to host powdery mildew fungi (*E. cichoracearum*) and non-host powdery mildew fungi (*Bgh*)^12^. In the present study, we isolated and identified a Qa-SNARE group gene, *MdSYP121*, from Fuji apple (Supplementary Fig. S1). The results of a series of genomic, genetic and transgenic experiments suggested that *MdSYP121* plays an important role in *B. dothidea* resistance in apple. An SNARE complex was also identified and may play an important role in pathogen resistance by affecting oxidation–reduction processes in apple following *B. dothidea* infection.

SNAREs proteins function as mediators of fusion between vesicular and target membranes^3^, and SYP121 syntaxin participates in vesicle fusion processes^33^. In Arabidopsis, SYP121 is a PM-localised protein^10^. Our analysis of the subcellular location revealed that MdSYP121 is located in the cell membrane (Fig. 1), indicating that the gene may function in membrane fusion and vesicular transport.

In plants, SA is an essential signalling molecule that induces SAR and is implicated in resistance to pathogens^34^. Many studies have shown that *EDS1* (*enhanced disease susceptibility 1*), *PAD4* (*phytoalexin-deficient 4*) and *PAL* (*Phenylalanine ammonia-lyase*) play important roles in SA biosynthesis^35,36^. *EDS1* and *PAD4* specifically promote the expression of principal SA biosynthetic enzyme gene *ICS1* (*Isochorismate synthase 1*)^35,37^. *PR* genes and *NPR1* are the marker genes of the SA signalling pathway^38^. The expression level of *PR* genes indicates the activity of SA signalling^39^. *NPR1* is considered as a positive regulator of SA-mediated plant immune responses, and *AtNPR1* is considered as a key regulator of SAR^40^. In the Arabidopsis syntaxin double mutant *syp121-1 syp122-1*, the SA level is dramatically elevated, resulting in necrosis and dwarfism. Interference of the SA signalling pathway in *syp121-1 syp122-1* mutants partially rescues the necrotic and dwarfed phenotype^18^. In the present study, silencing *MdSYP121* in ‘Gala’ apple plants lead to a necrotic and dwarfed phenotype (Supplementary Fig. S3), which is similar to the Arabidopsis syntaxin double mutant. We speculated that *MdSYP121* may play a negative role in SA signalling pathway. Due to physiological and biochemical changes in cultured cells and tissues infected by pathogens^41^, plant tissue culture systems can be used as model systems to study the plant defence responses to pathogenic bacteria^42,43^. In recent years, apple calli have been used as model experimental materials to study gene function. An et al. used *Agrobacterium*-mediated transformation to obtain transgenic apple calli overexpressing *MdHY5* to further investigate the functions of *MdHY5* in apple anthocyanin accumulation and nitrate assimilation^44^. Wang et al. reported that the roles of *MYB12* and *MYB22* in apple flavonoid biosynthesis were identified by OE of the genes in apple callus^22^. In this study, apple calli were used to further study the function of *MdSYP121* in response to *B. dothidea*. Based on our results, we noticed that silencing *MdSYP121* could increase the disease resistance to *B. dothidea*, while OE of the gene decreases resistance to *B. dothidea*. The expression of SA synthesis-related genes (*EDS1*, *PAD4* and *PAL*) and SA signalling pathway genes (*PR1*, *PR5* and *NPR1*) were highly elevated in the MdSYP121-RNAi lines after *B. dothidea* inoculation compared with the Vec line, and they were lower in the MdSYP121-OE lines than in the Vec line (Figs. 2 and 3). The results indicated that, by regulating the biosynthesis of SA and/or the activity of SA signalling, *MdSYP121* plays important roles in apple resistance to *B. dothidea*.

To better investigate the molecular mechanism of *MdSYP121* in regulating resistance to *B. dothidea*, RNA-Seq was used to study the expression levels of the genes before and after inoculation. Based on the results of RNA-Seq and the qRT-PCR assays, many genes associated with biotic stress were upregulated in the RNAi lines. The oxidation–reduction process (GO:0055114) and oxidoreductase activity (GO:0016491) constituted the major enriched GO terms in the upregulated group of genes (Fig. 4c). Most of the upregulated genes, including *APX* (MDP0000169497), *CAT* (MDP0000147628), *POD* (MDP0000611163) and *GST* (MDP0000300208), encoded proteins related to the synthesis of oxidoreductase. Enzymatic antioxidants such as POD and CAT are involved in scavenging H_2_O_2_ in living cells^45^. PODs are α-helical heme-containing proteins and play important roles in scavenging late massive reactive oxygen species (ROS) during plant–pathogen interactions. POD and CAT have prevailing functions in basal resistance and lignification against *Alternaria tenuissima* and are key resistance markers in potato^46^. Generation of ROS is among the earliest plant defence responses to various biotic stresses. ROS can enhance the hypersensitive response (HR) or act as secondary messengers in resistance mechanisms, leading to the upregulation of defence-related genes and interactions with other signalling molecules^47^. Previous studies have shown that SA pathway interacts with ROS in stressed plants^48^. *EDS1* plays an important role during oxidative stress caused by the release of singlet oxygen^49^. And *PAD4* also involves in the integrated regulation of ROS homoeostasis^50^. The pathogen *B. dothidea* causes cankers characterised by the collapse of cells and discoloured areas; these cankers alter cellular regulatory processes and increase the production of ROS. In this study, the results of *B. dothidea* inoculation assays suggested that the MdSYP121-RNAi lines displayed better tolerance than the Vec lines (Fig. 2). Higher expression levels of SA pathway-related genes and higher activities of APX, CAT, POD and GST were observed in the MdSYP121-RNAi lines than those in the Vec lines (Figs. 2 and 5). Based on our results, we speculated that *MdSYP121* influence disease resistance to *B. dothidea* by regulating the interaction of SA pathway and oxidation–reduction process.

The *Arabidopsis thaliana* SYP121 syntaxin resides in the PM and was previously shown to act together with its partner SNAREs, the adaptor protein SNAP33 and endomembrane-anchored VAMP721/722 to form a ternary SYP121-SNAP33-VAMP721/VAMP722 SNARE complex, which is required for in the execution of secretory immune responses against powdery mildew fungi^12^. The results of the analysis of the subcellular localisation of MdSYP121 and MdSNAP33 revealed that green fluorescence was detected in the cell membrane, indicating that MdSYP121 and MdSNAP33 are located in the PM and may function together in vesicular transport and membrane fusion (Figs. 1 and 6). In apple, MdSYP121 and MdSNAP33 may also function in disease resistance by forming a complex. The ternary SNARE complexes are SDS-resistant but heat sensitive^12,29^. The abundance of SYP121-containing SNARE complexes were examined in Vec and MdSYP121-OE lines by comparing band disappearances in response to boiling in an immunoblot solution containing anti-GST antibody (Fig. 6c). However, we detected the presence of the complex in both Vec and MdSYP121-OE lines, which is consistent with the higher levels of MdSYP121 monomers in the MdSYP121-OE lines. Previous studies have shown that the formation of SYP121-dependent ternary SNARE complexes is critical for plant pre-invasive resistance to powdery mildew fungi^12^. The MdSYP121 complex identified in our research may participate in resistance to *B. dothidea*.

In conclusion, *MdSYP121* is involved in balancing penetration resistance and regulating SA-mediated defences and oxidation–reduction processes; both of these functions are important in apple ring rot resistance. The results show that *MdSYP121* plays a central regulatory role in resistance against *B. dothidea* penetration in apple. Our results imply a potential function of *MdSYP121* in apple resistance to *B. dothidea*. This knowledge concerning defence mechanisms involved in *B. dothidea* resistance could be useful in breeding programmes aiming to introduce apple genotypes that exhibit high levels of immunity against this destructive fungus. This knowledge could also promote the breeding of new cultivars that display enhanced disease resistance capabilities against this devastating disease.

## Electronic supplementary material

Supplement Fig. S1-S5 and Table S1-S2(DOCX 20779 kb)
