# Supplementary material for: Activation of disease resistance against Botryosphaeria dothidea by downregulating the expression of MdSYP121 in apple
Source: Hortic Res. 2018 May 1;5:24. doi: 10.1038/s41438-018-0030-5 (PMC5928070; doi:10.1038/s41438-018-0030-5)
Supplement: Supplementary file 1 — Supplement Fig. S1-S5 and Table S1-S2(DOCX 20779 kb) [file 41438_2018_30_MOESM1_ESM.docx]

**
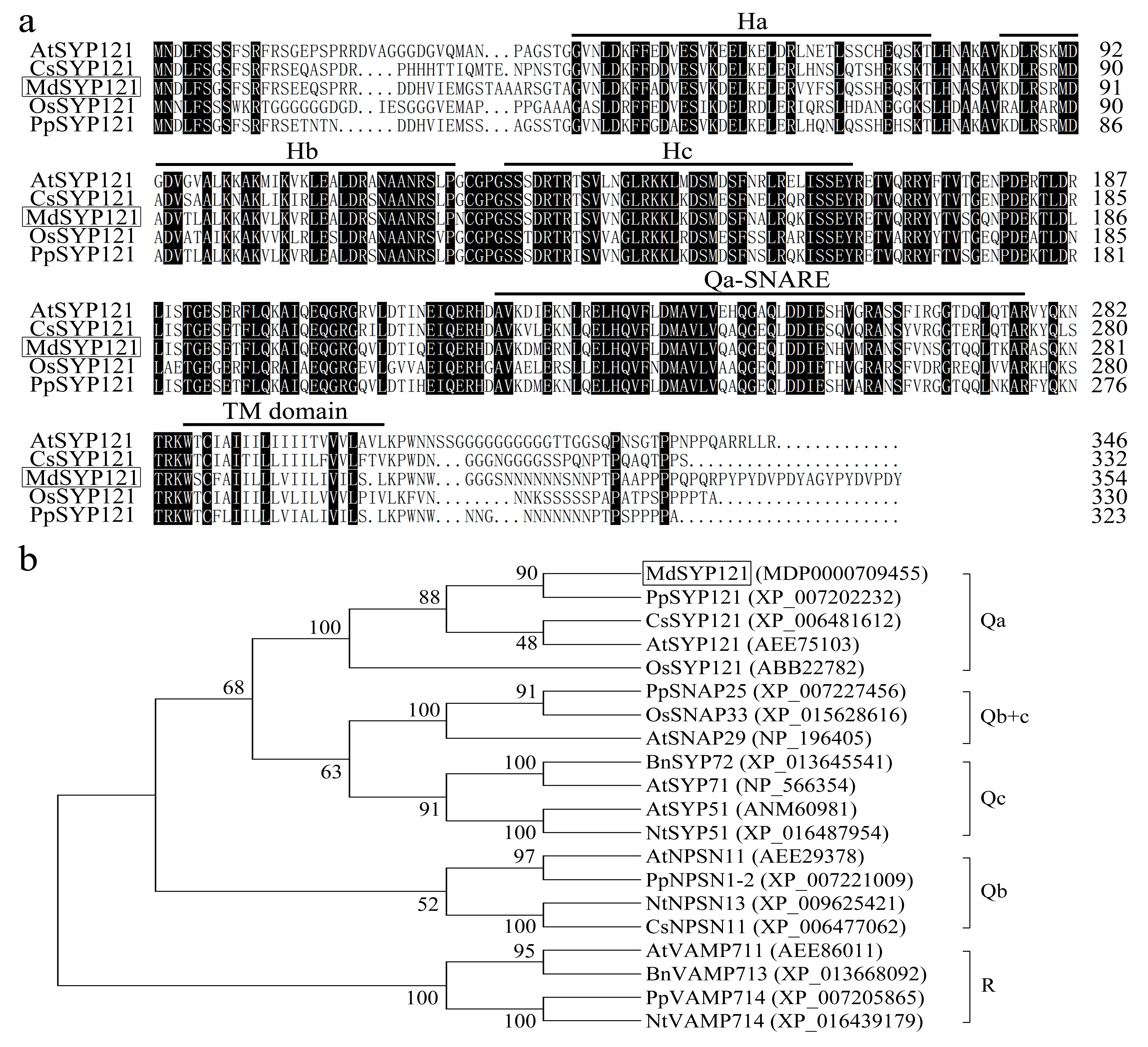
**

**Fig. S1.** Characterization and sequence analysis of MdSYP121. (**a**). Multiple amino acid sequence alignment of MdSYP121, AtSYP121, CsSYP121, OsSYP121 and PpSYP121. The conserved kinase domains include Ha-, Hb- and Hc-SNAREs; Qa-SNARE and the transmembrane domain (TM domain) are labelled with a horizontal line. (**b**). Dendrogram analysis. Phylogenetic relationships between MdSYP121 and other plant SNARE proteins. The numbers above and below the branches indicate bootstrap values (>50%) from 1,000 replicates. Qa, Qb, Qc, Qb+c and R indicate SNARE groups.

**
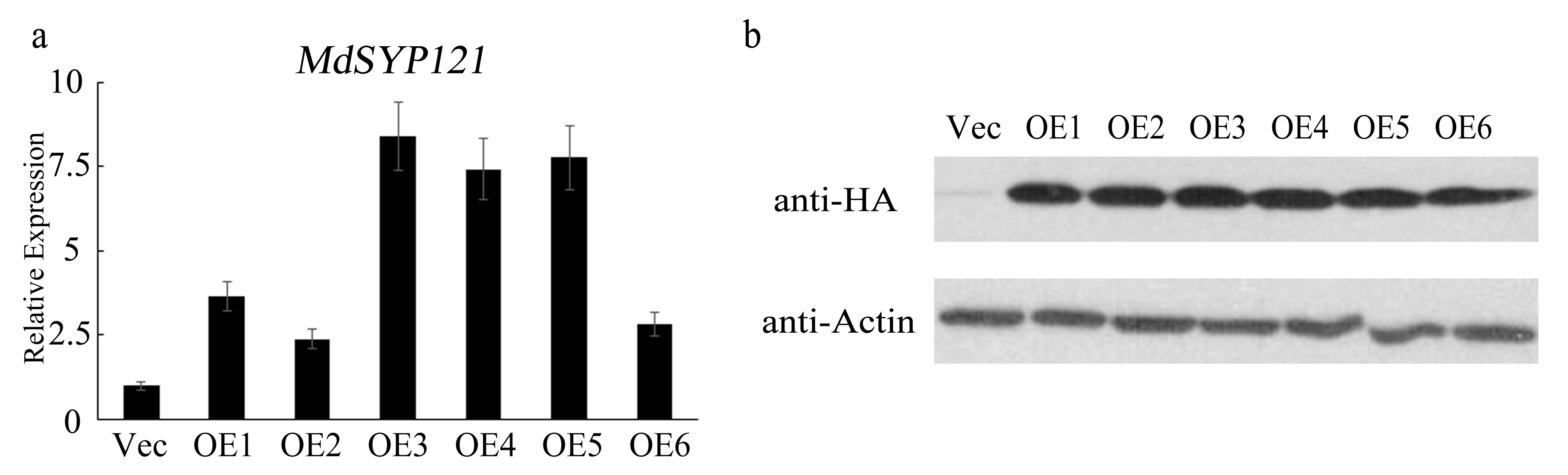
**

**Fig. S2.** MdSYP121 mRNA relative expression and protein levels in overexpression transgenic calli lines. (**a**). The expression level of *MdSYP121* in overexpression transgenic calli lines. The data are the mean values ±SEs of three independent experiments. (**b**). The protein level of MdSYP121 in overexpression transgenic calli lines.

**
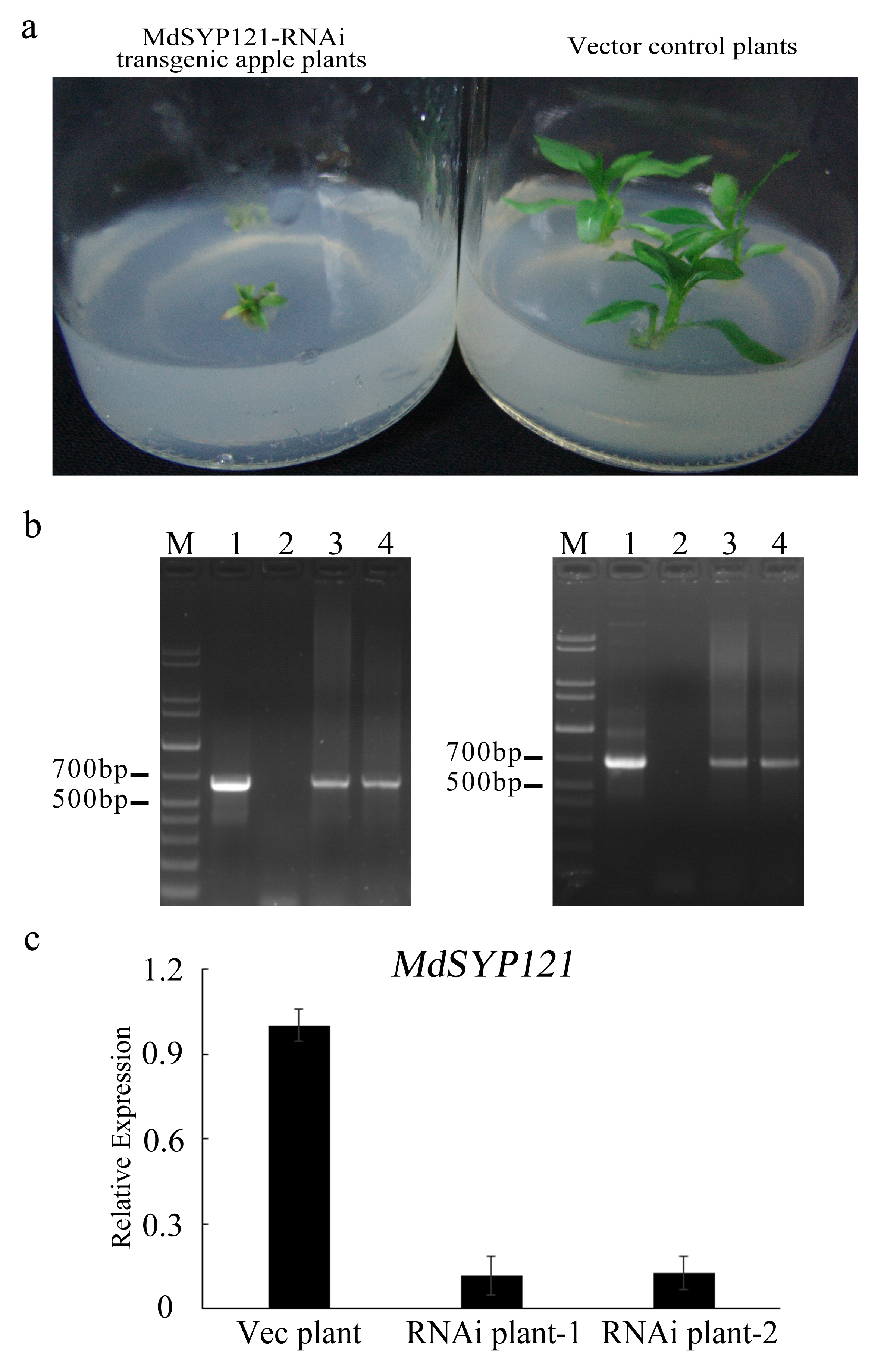
**

**Fig. S3.** Representative phenotypes of transgenic apple plants and identification of MdSYP121-RNAi transgenic plants. (**a**). Representative phenotypes of transgenic apple plants and Vector control plants. (**b**). The RNAi fragment of *MdSYP121* was integrated into the genome of ‘Gala’ plants. Left: The RNAi fragment of *MdSYP121* was detected using MdSYP121-RNAi-1-5 and pCB302-RNAi-1-3 primers; Right: The RNAi fragment of *MdSYP121* was detected using MdSYP121-RNAi-2-5 and pCB302-RNAi-2-3 primers. M: maker; 1: Positive vector control; 2: Vec plant; 3: RNAi plant-1; 4: RNAi plant-2. (**c**). The expression level of *MdSYP121* in RNAi transgenic plants. The data are the mean values ±SEs of three independent experiments.

**
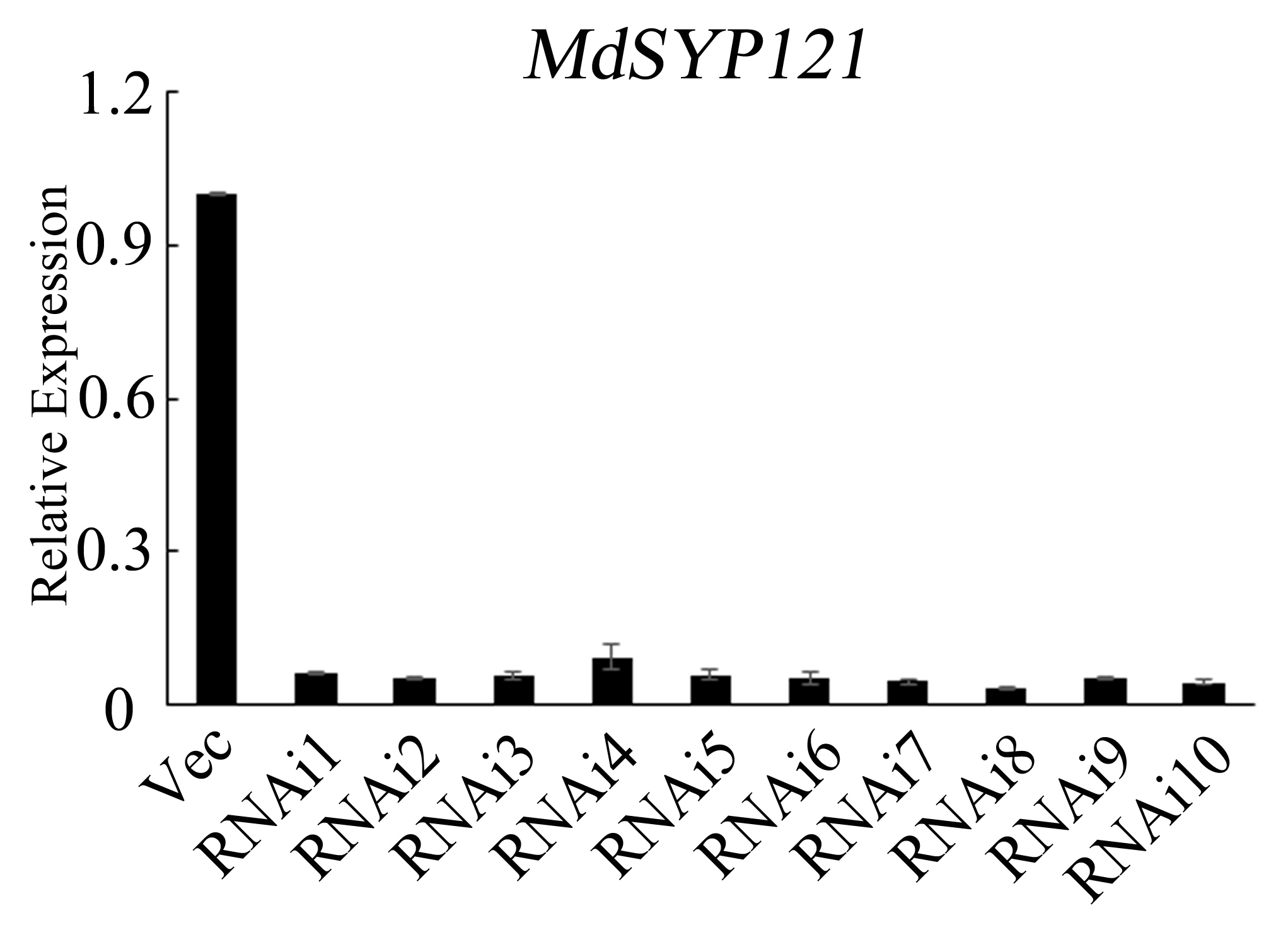
**

**Fig. S4**. Expression level of *MdSYP121* in RNAi transgenic calli lines. The expression level of *MdSYP121* was detected in ten different RNAi calli lines. The data are the mean values ±SEs of three independent experiments.


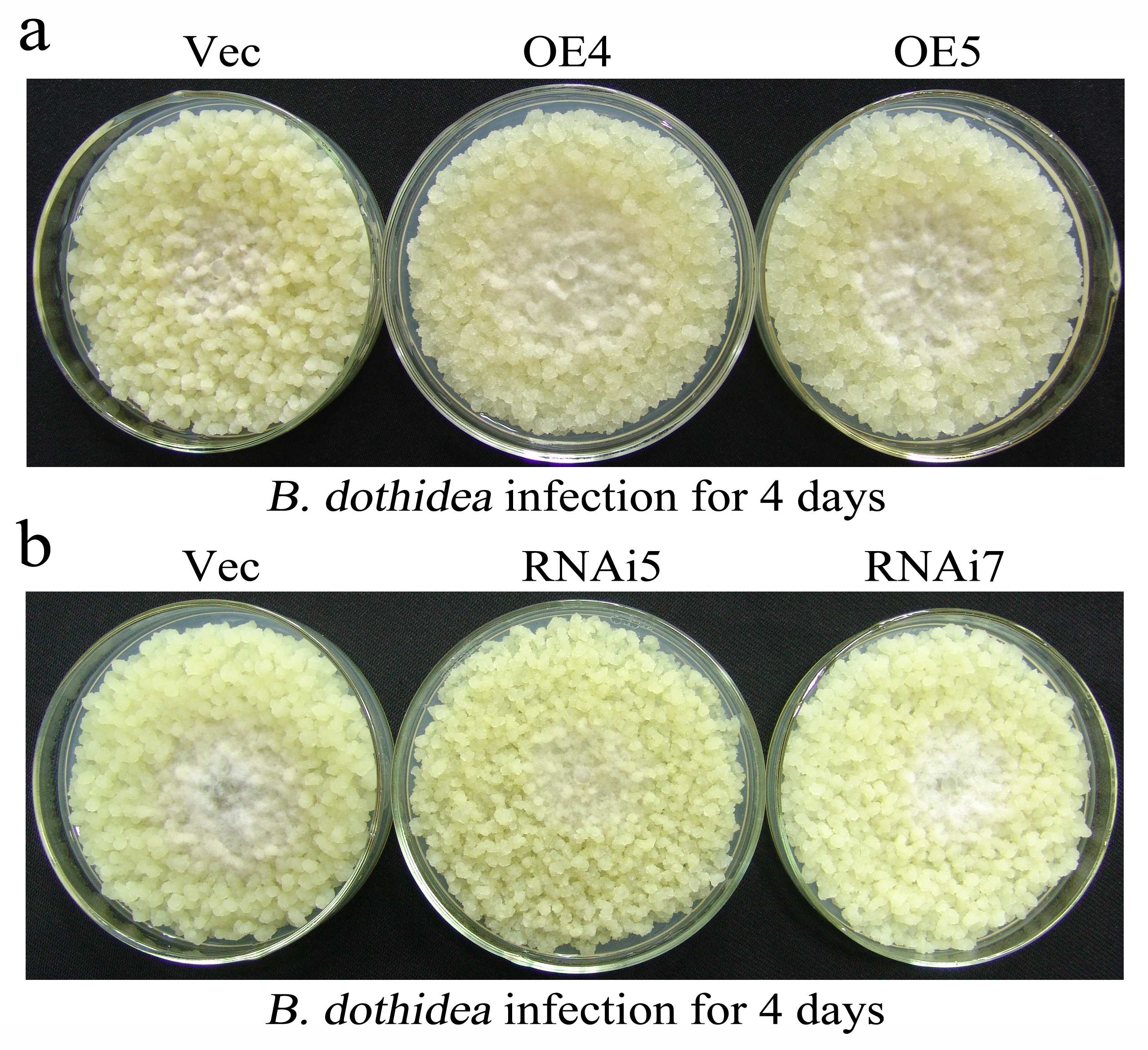


**Fig. S5.** Representative phenotypes of calli lines with *B. dothidea* infection. (**a**). Representative phenotypes of Vec and MdSYP121-OE calli lines with *B. dothidea* infection for 4 days. (**b**). Representative phenotypes of Vec and MdSYP121-RNAi calli lines with *B. dothidea* infection for 4 days.

**Table S1.** Oligonucleotide primers used in gene cloning, vectors construction and qRT-PCR.

| Primer | Primer sequence (5'→3') |
| --- | --- |
| Vector construct primers |  |
| MdSYP121-HA-5 | CGGGATCCATGAACGATTTGTTCTCCGGCTCCT |
| MdSYP121-HA-3 | AAGGCCTAGCGTAGTCTGGAACGTCGT |
| MdSNAP33-GST-5 | CCGCGTGGATCCCCGGAATTCATGTTCGGTTTAAAGAAGTCTCCTTT |
| MdSNAP33-GST-3 | GTCACGATGCGGCCGCTCGAGCTACTTTCCGAGCAAGCGACG |
| MdSYP121-RNAi-1-5  MdSYP121-RNAi-1-3  MdSYP121-RNAi-2-5  MdSYP121-RNAi-2-3  pCB302-RNAi-1-3  pCB302-RNAi-2-3  Primers used on qRT-PCR | CGGGATCCGTCAACCTCGACAAGTTCTTC  CCATCGATCTTGCTCCTGTATTGCCTT  CCGCTCGAGGTCAACCTCGACAAGTTCTTC  GGGGTACCCTTGCTCCTGTATTGCCTT  GGTAACATGATAGATCATGTC  CTTCGTCTTACACATCACTTGTC |
| MdSYP121-Q-5  MdSYP121-Q-3  Mdactin-F  Mdactin-R  BiFC vector construct primers  MdSYP121-C-5  MdSYP121-C-3  MdSNAP33-N-5  MdSNAP33-N-3 | GCGGTTCCGGAGCGAGGAGCAG  CGTCCTTGACGGACTCGACGTCGGC  TACTCAGCTTTGGCAATCCACATC  TGACCGAATGAGCAAGGAAATTACT  TCTAGAATGAACGATTTGTTCTCCGGC  GGTACCAGCGTAGTCTGGAACGTCGT  GGATCCATGTTCGGTTTAAAGAAGTCTCC  CTCGAGCTTTCCGAGCAAGCGACG |

**Table S2.** Oligonucleotide primers used in qRT-PCR.

| Primer | Primer sequence (5'→3') |
| --- | --- |
| MdPR1-5 | CGATTGCAGTGAAGATGTGGGTTT |
| MdPR1-3 | CCATCAAACCCAACACAATGTCCTTA |
| MdPR5-5 | CAAGTGAAAGCGGCTGATGGGAG |
| MdPR5-3 | GTCCACCACTGCAGGTAAATGTGC |
| MdEDS1-5  MdEDS1-3  MdNPR1-5  MdNPR1-3  MdPAD4-5  MdPAD4-3  MdPAL-5  MdPAL-3  MdPOD-5  MdPOD-3  MdAPX-5  MdAPX-3  MdGST-5  MdGST-3  MdCAT-5  MdCAT-3 | GAAACTGAATGGGCAACGATC  CATAAGGCTCGGTACTCTTCG  GATCTTACGCTGGATGGGAGGAAAG  GAAAGCGAGGCTTCTCCAAGCAAC  GTGAGCAACTGGGGTACTATGACG  GGAGTTTGTAAAACTGGGAAGCATTTAC  CCTCAAGATTCTGCGAGAAGGATCTC  GCTCAACAAGCACTTGCCTCAGTT  CGGGCAAGATGTTCAACTTTCGTTC  CATCGCGGGTGGACTGGTCAAG  GTTGTCGCTGTCGAGGTCACTGG  CCACAATGTCCTTATCAGACAGACCC  TCGAGGTGACACAGTTGGATGGC  CAGCAATGTACCCTCCCCCGAG  GTCAAGTGTTTGTTGGAGGATGAGG  CAGGCCAGGTCTTAGTAACATCAAGT |
